# Supplementary material for: Brillouin zone folding driven bound states in the continuum
Source: Nat Commun. 2023 May 17;14:2811. doi: 10.1038/s41467-023-38367-y (PMC10192215; doi:10.1038/s41467-023-38367-y)
Supplement: Supplementary file 1 — Supplementary Information [file 41467_2023_38367_MOESM1_ESM.pdf]

Supplementary Information

Brillouin Zone Folding Driven Bound States in the Continuum

Wenhao Wang[1,2,3], Yogesh Kumar Srivastava[2,3], Thomas CaiWei Tan[2,3], Zhiming Wang[1,*] and Ranjan Singh[2,3,*]

[1]*Institute of Fundamental and Frontier Sciences, University of Electronic Science and Technology of China, Chengdu 610054, China*

[2]*Division of Physics and Applied Physics, School of Physical and Mathematical Sciences, Nanyang Technological University, Singapore 637371, Singapore*

[3]*Centre for Disruptive Photonic Technologies, The Photonics Institute, Nanyang Technological University, Singapore 637371, Singapore*

[*]Corresponding Authors: Zhiming Wang: zhmwang@uestc.edu.cn, Ranjan Singh: ranjans@ntu.edu.sg

Supplementary Section S1: Scaling rules of the *Q* factors of GRs, BICs, and BZF-BICs

Guided resonances (GRs) are formed from guided modes due to periodic perturbation-induced band folding. The Q factors of these leaky modes are related to the perturbation magnitude, α. The relationship between them is shown in Eq. 1 and reproduced here as:

$$Q = Q_0 / \alpha^2, \qquad \text{(S1)}$$

which can be derived from a combination of perturbation theory and temporal coupled mode theory[1-2]. As shown in Fig. S1a, the Q factor of GRs TE_{11}, TE_{31}, and TE_{41} modes at Γ point is well fitted by Eq. S1. From the enlarged Q factor distribution on the right panel of Fig. S1a, we could see that Eq. S1 is valid when α is smaller than 0.37 for TE_{11} and TE_{31} modes and 0.23 for TE_{41} mode. The evolution of symmetry-protected BICs' Q factor in the momentum space follows the rules of[3-4]

$$Q = Q_0 / k^2, \qquad \text{(S2)}$$

As shown in Fig. S1b, the Q factor of TE_{40} mode for a gap-perturbed PhC ($\alpha = 0.0167$, $\Delta L = 1$ μm) is well fitted by Eq. S2 when k is smaller than $0.08 \cdot 2\pi/a_1$.

Since BZF-BIC arises from band folding, the Q factor of quasi-BZF-BIC should also follow the scaling rule of Eq. S1. The left panel of Fig. S1c shows the Q factor of quasi-BZF-BIC TE_{21} and TE_{51} modes at $(k_x, k_y) = (0.01 \cdot 2\pi/a_1, 0)$. To exclude the effect of k on the fitting constant Q_0, we use a modified version of Eq. S1 to fit the Q factor:

$$Q = Q_0 / (0.005^2 \cdot \alpha^2), \qquad \text{(S3)}$$

As shown in the middle panel of Fig.S1c, the Q factors of TE_{21} and TE_{51} modes are fitted well by Eq. S1a when α is smaller than 0.167 for both TE_{21} and TE_{51} modes. The fitted Q_0 is 9.02 and 0.185 for TE_{21} and TE_{51} modes, respectively. In addition, since BZF-BIC is symmetry-protected BIC, its Q factor should also follow the scaling rule of Eq. S2 in the momentum space. The right panel of Fig. S1c shows the Q factor of quasi-BZF-BIC TE_{21} and TE_{51} modes for a gap-perturbed PhC ($\alpha = 0.0167$, $\Delta L = 1$ μm). They are well fitted respectively by

$$Q = 9.02 / (0.0167^2 \cdot k^2), \qquad \text{(S4)}$$

$$Q = 0.185 / (0.0167^2 \cdot k^2), \qquad \text{(S5)}$$

when k is smaller than $0.2 \cdot 2\pi/a_1$ for TE_{21} mode and $0.045 \cdot 2\pi/a_1$ for TE_{51} mode. From the above mathematical relations, it is easy to conclude that the Q factor BZF-BIC should follow both the rules of Eq. S1 and S2, that is

$$Q = Q_0 / (\alpha^2 k^2). \qquad \text{(S6)}$$

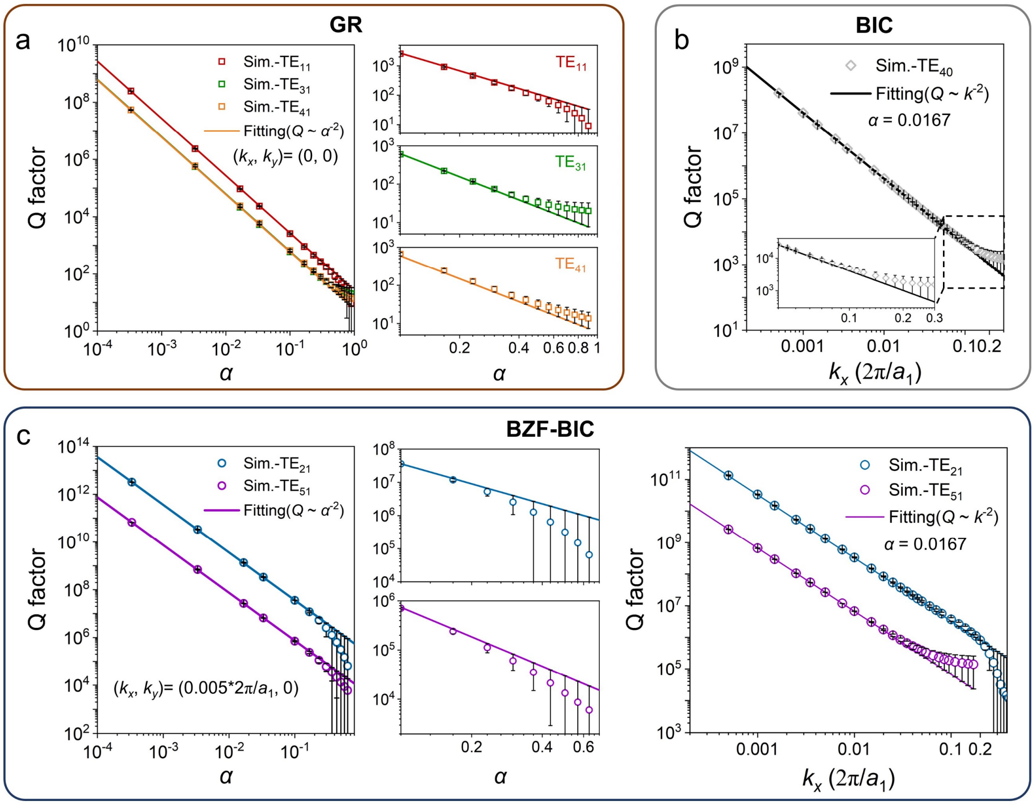

Fig. S1. Scaling rules of the Q factors of GRs, BICs, and BZF-BICs. a, Simulated Q factors of GRs TE_{11}, TE_{31}, and TE_{41} modes at Γ point. They are fitted by $Q \propto 1/\alpha^2$ (solid lines). The enlarged Q factor distributions at large α values are shown on the right panel. b, Simulated Q factor of TE_{40} mode for a gap-perturbed PhC ($\alpha = 0.0167$, $\Delta L = 1$ μm). It is fitted by $Q \propto 1/k^2$. Inset shows the enlarged Q factor distribution at large k_x values. c, Simulated Q factors of quasi-BZF-BIC TE_{21} and TE_{51} modes for different α values at $(k_x, k_y) = (0.01 \cdot 2\pi/a_1, 0)$ (left and middle panels) and for different k_x values with a gap-perturbed PhC ($\alpha = 0.0167$, $\Delta L = 1$ μm) (right panel). They are fitted by $Q \propto 1/\alpha^2$ and $Q \propto 1/k^2$, respectively.

Supplementary Section S2: Characterization of disorder in fabricated samples

Samples were fabricated using the conventional UV photolithography process, followed by RIE and DRIE processes (see Methods in the main text). Fig. S2a shows the optical microscopy (OM) image of a gap-perturbed sample (ΔL = 18 μm). The measured diameter is 81.75 μm, which is 1.75 μm larger than the designed value of 80 μm. We then repeated the measurement over 100 air holes with 3 samples having

different ΔL, and summarized the counts of deviations of radius, dr (Fig. S2b). The average dr is estimated to be 0.80 μm. Similarly, the average deviation of the position of air holes in the x direction dx, and y direction dy are estimated to be 0.62 μm and 0.70 μm, respectively. These values were used in the simulations to study the effect of disorders on eigenmode's Q factor.

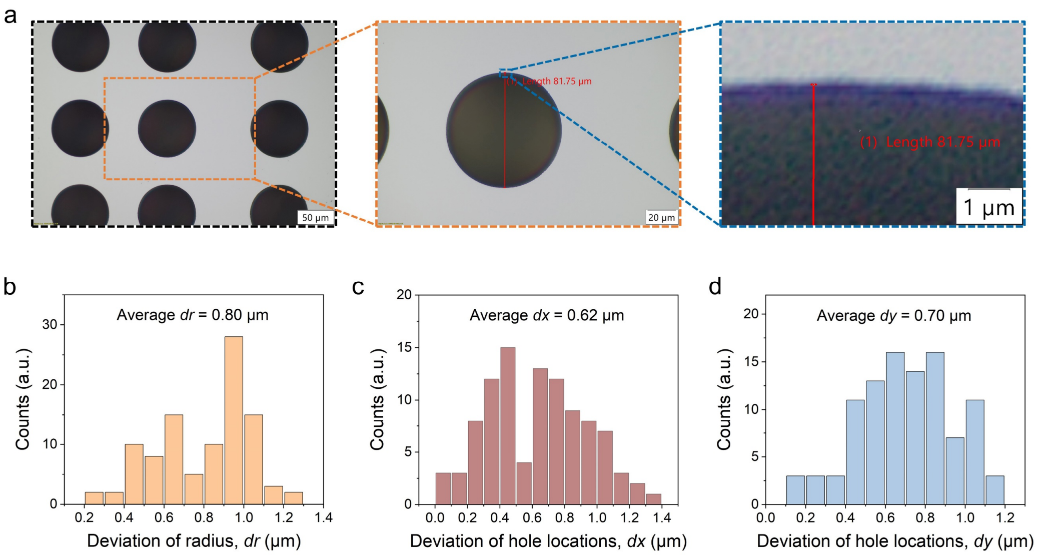

Fig. S2. Disorders of fabricated samples. a, Optical microscopy (OM) image of the fabricated gap-perturbed PhC sample (ΔL = 18 μm). Distributions of the deviations of b, air hole's radius dr and locations c, dx and d, dy over 100 air holes with 3 samples having different ΔL.

Supplementary Section S3: Far-field polarization maps of eigenmodes under gap perturbation and radius perturbation

The far-field polarizations of eigenmodes are obtained by spatially averaging the electric field on any horizontal plane outside the THz-PhC slab, following the method mentioned in ref [5]. As shown in Fig. S3a, when a gap perturbation (ΔL = 1 μm) is introduced, the far-field polarization maps of GRs $TE_{11,\Gamma}$, $TE_{31,\Gamma}$, and $TE_{41,\Gamma}$ modes show trivial distribution (no vortex in the center of Brillouin zone). For BZF-BIC $TE_{21,\Gamma}$

and BIC $TE_{40,\Gamma}$ modes, the polarization ellipse winds around the Γ point and the angle between its major axis and *x*-axis change 2π after traveling around a simple closed path in the counterclockwise direction. According to Eq. 3, these BICs carry +1 topological charge. For BZF-BIC $TE_{51,\Gamma}$ mode, the angle changes -2π and it hence carries -1 topological charge. The allowed topological charges at Γ point for single degenerated bands can be determined by checking the field eigenvalues of the rotational symmetry of the system[5]. By using structures with high rotational symmetry, higher order topological charges can be found. For example, topological charge of -2 has been observed at structures having C_6 symmetry[6-7].

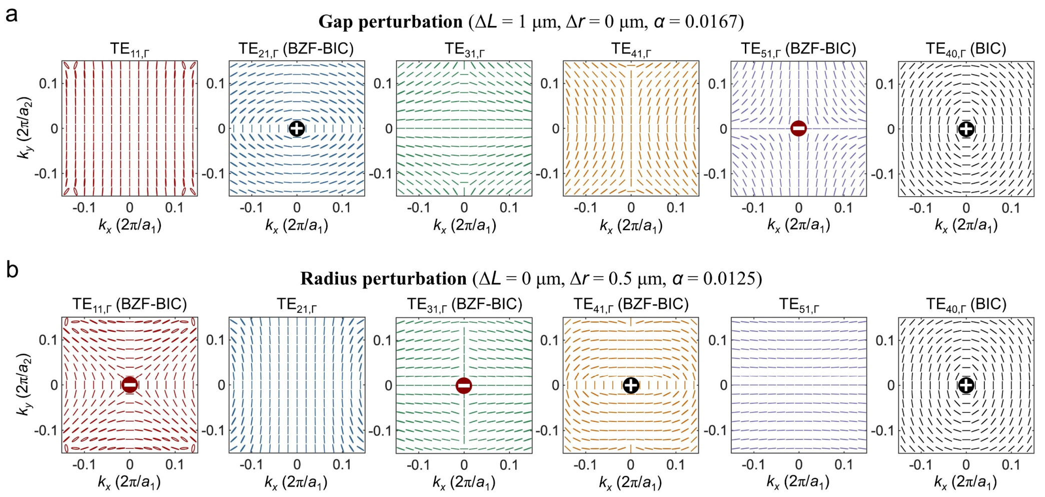

Fig. S3. Far-field polarization maps of folded modes $TE_{11,\Gamma\sim51,\Gamma}$ and un-folded mode $TE_{40,\Gamma}$ under a, gap perturbation and b, radius perturbation, respectively.

However, when a radius perturbation (Δr = 0.5 μm) is introduced, the polarization features of the original GRs and BZF-BICs are switched: the original BZF-BICs $TE_{21,\Gamma}$ and $TE_{51,\Gamma}$ modes are now GRs and their polarization maps show trivial distribution; the original GRs $TE_{11,\Gamma}$, $TE_{31,\Gamma}$, and $TE_{41,\Gamma}$ modes become BZF-BICs and their

polarization maps present vortices feature. Interestingly, the polarization map of BIC $TE_{40,\Gamma}$ mode nearly does not change. This is because $TE_{40,\Gamma}$ mode is formed without band folding and hence is robust to the periodic perturbation.

Supplementary Section S4: Simulation of *Q* factors for disordered PhC

For actual samples, structural disorders happen arbitrarily, breaking the ideal periodicity of perfect PhC. To study the *Q* factors of disordered PhC, we assume that the structure is still periodic in an $N \times N$ supercell. The supercell can be regarded as a realistic large-area sample when *N* is large enough. However, the size of the simulation model increases dramatically with *N*, and the simulation time becomes unrealistically long for a large *N*. To make better trade-off between accuracy and efficiency, we first studied the effect of supercell size on the *Q* factor. Fig. S4a shows the simulated *Q* factor of TE_{21} mode using unit cell without disorder and disordered supercells. Gap perturbation ΔL = 1 μm is applied for all the structures. The *Q* factor drops as the supercell size *N* increases, especially in the momentum space near Γ point. The increased radiation loss arises from the enhancement of the coupling between folded mode TE_{21} and leaky channels in the continuum and the coupling between TE_{21} and other folded modes in different *k* values. It can be noted that the *Q* factors are similar for disordered 8×8 and 12×12 supercells, suggesting that an 8×8 supercell is enough and reliable to study the *Q* factors of disordered PhC.

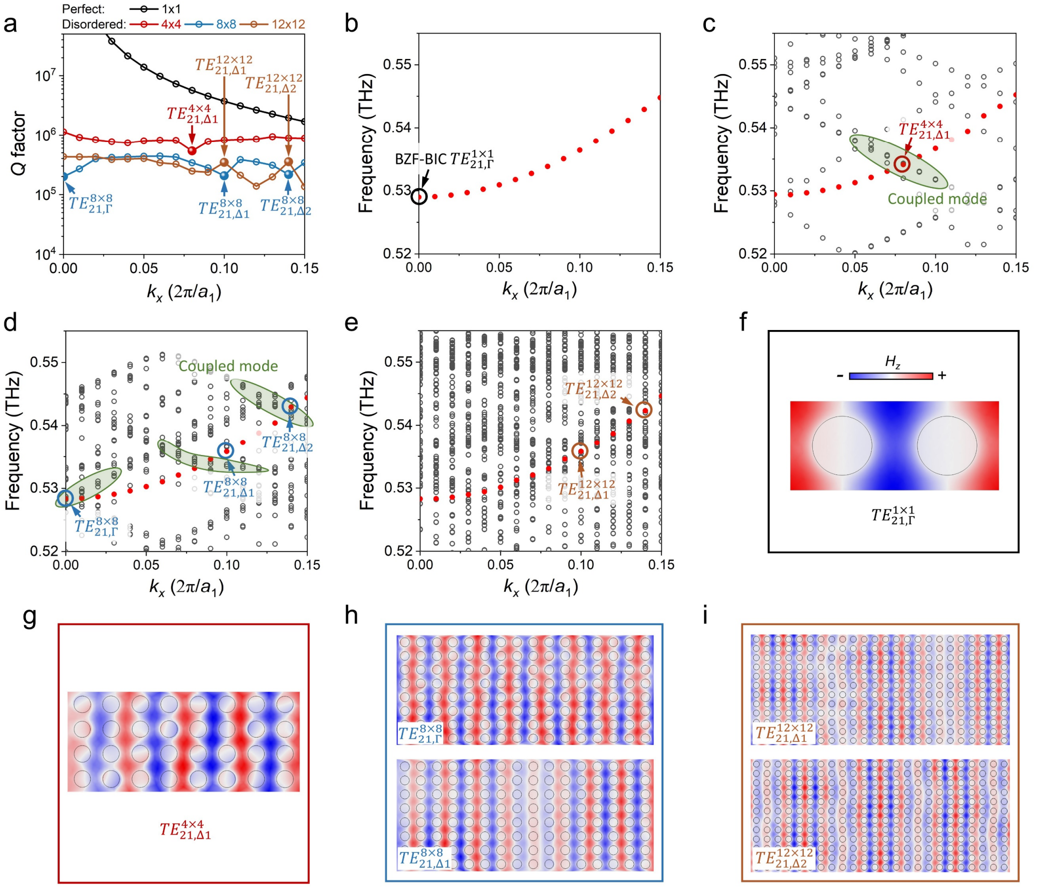

Fig. S4. The effect of supercell size on the Q factors of disordered PhCs. a, Simulated Q factors of TE_{21} mode using unit cell without disorder and using disordered supercells. Gap perturbation ΔL = 1 μm is applied for all the structures. Simulated eigenmodes supported in b, a unit cell, c, 4×4, d, 8×8, and e, 12×12 supercells. Simulated magnetic field profiles H_z of f, $TE_{21,\Gamma}^{1\times1}$, g, $TE_{21,\Delta1}^{4\times4}$, h, $TE_{21,\Gamma}^{8\times8}$ and $TE_{21,\Delta1}^{8\times8}$, and i, $TE_{21,\Delta1}^{12\times12}$ and $TE_{21,\Delta2}^{12\times12}$ eigenmodes.

We also notice that there are dips and peaks in the Q factors of disordered PhCs, which changes with the size of the supercell. The fluctuation of the Q factors comes from the coupling with other folded modes induced by the periodic boundaries. As shown in Fig. S4b, TE_{21} band (red dots) is very clean in the band structure of unit cell perfect PhC. The H_z field distribution of BZF-BIC $TE_{21,\Gamma}^{1\times1}$ mode shows perfect even symmetry under C_2 operation regarding the middle of air holes. However, the number of supported eigenmodes increases 15 times in the 4×4 supercell compared to that in the unit cell

since the first Brillouin zone (FBZ) is squeezed to its previous 1/16 and modes previously located outside are folded into the new FBZ. Due to the introduction of disorder, the new folded modes couple with TE_{21} mode and affect its radiation loss (Fig. S4c). The H_z field distribution at the Q factor dip, $TE_{21,\Delta 1}^{4\times 4}$ mode, shows a perturbed feature and presents quasi-even symmetry under C_2 operation regarding the center of supercell (Fig. S4g). As the supercell size increases, the FBZ is further squeezed and modes in a larger portion of the previous momentum space are folded into the new FBZ and coupled with TE_{21} mode.

Supplementary Section S5: Point group symmetries analysis

The unperturbed, gap-perturbed, and radius-perturbed PhCs have an in-plane point group symmetry $C_{2v} = \{E, C_2, \sigma_x, \sigma_y\}$, where the symmetry operations are illustrated in Fig. S5. For the unperturbed PhC, there are two types of structural high symmetry points: the center of the air holes and the middle portion of adjacent air holes, respectively represented by the yellow and green dots. However, the radius-perturbed and gap-perturbed PhCs only have the structural high symmetry point in the center of the air holes and the middle portion of adjacent air holes, respectively. For the C_{2v} point groups, the irreducible representations and their characters are listed in Table S1.

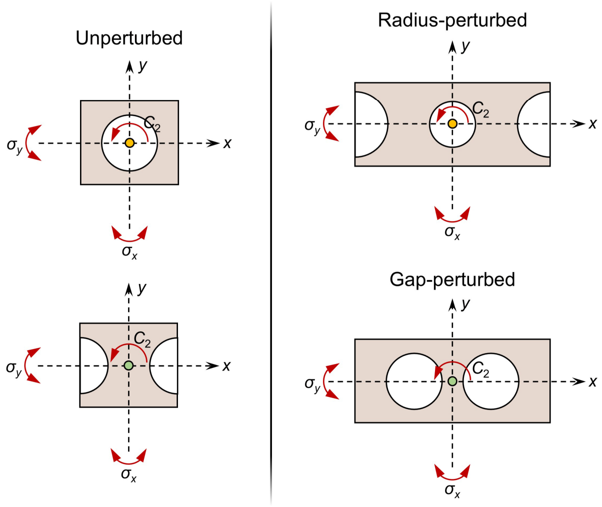

Fig. S5. Symmetry operations for the rectangular array of the unperturbed, radius-perturbed, and gap-perturbed PhCs.

Table S1. The character table for the C_{2v} point group

C_{2v}	E	C_2	σ_y	σ_x
A_1	1[a]	1	1	1
A_2	1	1	-1[a]	-1
B_1	1	-1	1	-1
B_2	1	-1	-1	1

[a]The number 1 and -1 indicate a symmetric and antisymmetric profile after applying symmetry operations, respectively.

According to the eigenmodes' field profile shown in Fig. 3, the irreducible representation of the modes is identified and summarized in Table S2. For the unperturbed PhCs, $TE_{4,\Gamma}$ mode has both A_1 irreducible representation regarding the center of the air holes and the middle portion of adjacent air holes. However, for all the modes in the X point ($TE_{1\sim5,X}$), they have different irreducible representations regarding different structural high symmetry points. When gap or radius perturbation is

introduced, the folded modes $TE_{(1\sim5)1,\Gamma}$ and unfolded mode $TE_{40,\Gamma}$ has the irreducible representation with respect to the remaining structural high symmetry point.

Table S2. The irreducible representations of eigenmodes in PhCs with and without perturbations

Modes	Without perturbation	Gap perturbation	Radius perturbation
	M[a] / C[a]	M	C
$TE_{4,\Gamma\,/\,40,\Gamma}$	A_1 / A_1	A_1	A_1
$TE_{1,X\,/\,11,\Gamma}$	B_1 / A_1	B_1	A_1
$TE_{2,X\,/\,21,\Gamma}$	A_1 / B_1	A_1	B_1
$TE_{3,X\,/\,31,\Gamma}$	B_2 / A_2	B_2	A_2
$TE_{4,X\,/\,41,\Gamma}$	B_1 / A_1	B_1	A_1
$TE_{5,X\,/\,51,\Gamma}$	A_2 / B_2	A_2	B_2

[a]M and C denote the high symmetry points of the structure at the middle of adjacent air holes and the center of air holes, respectively.

Supplementary Section S6: Excitation of TE eigenmodes with different polarized sources

The eigenmodes of every two-dimensional PhC can be classified into two distinct polarizations[8]: transverse electric (TE) modes, in which the electric field is confined to the *xy* plane (E_x, E_y, H_z), and transverse magnetic (TM) modes, in which the magnetic field is confined to the *xy* plane (H_x, H_y, E_z). Due to the finite thickness of PhC, the fields become mostly TE-like and TM-like when moving away from the mirror plane of the structure in the *z* direction. Fig. S6a shows the calculated TE band structure of gap-perturbed (ΔL = 30 μm, α = 0.5) PhC. For TE polarized plane wave (Fig. S6c), it carries both electric and magnetic field components in the *xy*-plane: (H_x, E_y, 0) for

normal incidence and (H_x, E_y, H_z) for oblique incidence. The situation is similar for TM polarization: (E_x, H_y, 0) for normal incidence and (E_x, H_y, E_z) for oblique incidence. The available eigenmodes that can be excited by a certain polarized light depend on the symmetry matching condition between the eigenmodes' field profiles and the excitation source[9]. Specifically, as shown in Fig. S6d, under the mirror reflection operation around the x axis, which changes y to $-y$ (noted as σ_y), the magnetic field profiles of $TE_{11,\Gamma}$, $TE_{21,\Gamma}$, $TE_{41,\Gamma}$, and $TE_{40,\Gamma}$ modes remain the same, showing an even feature. On the contrary, the magnetic field profiles of $TE_{31,\Gamma}$ and $TE_{51,\Gamma}$ modes present an odd feature under σ_y operation. Here σ_y is considered because when moving away from Γ to X point, the symmetry group changes from C_{2v} to C_{1h}, and only the representations E and σ_y retain[10]. Since the magnetic vector of TE (TM) polarized light is even (odd) under σ_y operation, TE_{11}, TE_{21}, TE_{41}, and TE_{40} (TE_{31} and TE_{51}) modes are excited and observed in the TE(TM)-polarized transmission spectra (Fig. S6b).

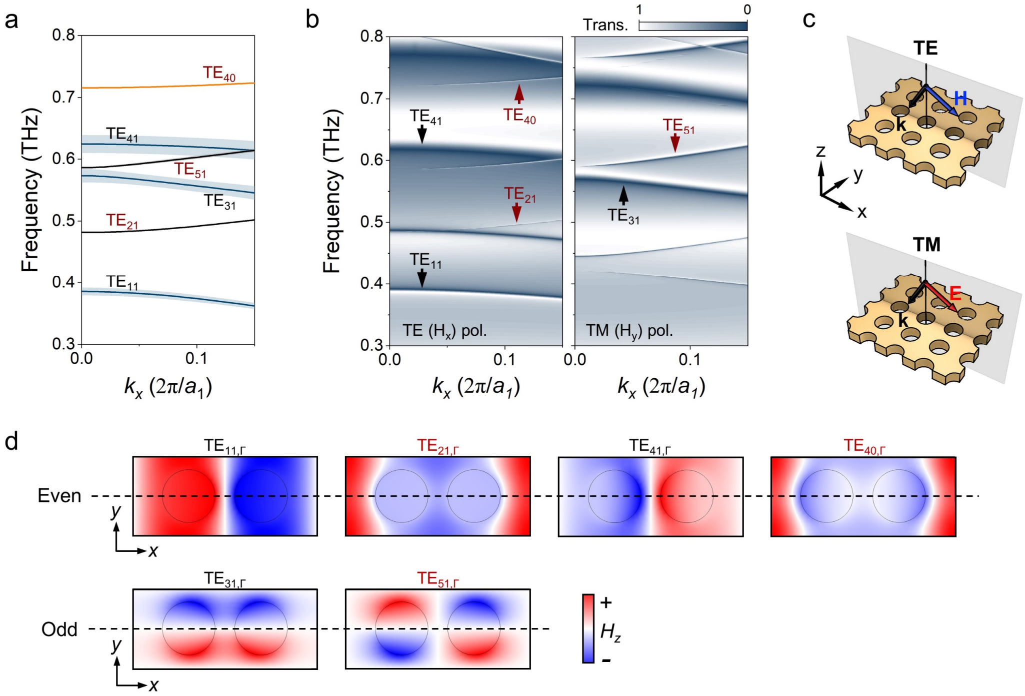

Fig. S6. Excitation of TE eigenmodes with different polarized sources. a, Calculated transverse electric (TE) band structure of gap-perturbed (ΔL = 30 μm, α = 0.5) PhCs. The blue, black, and yellow lines represent the bands having GRs, BZF-BICs, and BIC, respectively. The shaded blue area around the GRs shows the linewidth of the modes. b, Calculated angle-resolved transmission spectra of THz-PhC (ΔL = 30 μm) along the Γ-X direction under TE and TM polarizations. c, Schematics of the magnetic and electric vectors configurations of oblique incident TE and TM polarized light. d, Calculated magnetic field profiles H_z of eigenmodes at Γ point for gap-perturbed (ΔL = 30 μm) PhC.

Supplementary Section S7: Transmission spectra obtained by using fiber-based and ZnTe THz-TDSs.

In this work, two THz measurement systems, including fiber-based and ZnTe THz-TDSs, were used. To have a better understanding about the measurement performances of these two systems, here we compare the transmission spectra obtained by them. As shown in Fig. S7a and S7b, the measured transmission spectra are very similar using fiber-based and ZnTe THz-TDSs for both TE and TM polarizations. We extract the Q

factors of GRs TE_{11}, TE_{31}, and TE_{41} modes by fitting the transmittance spectra with Eqs. 4-6. As shown in Fig. S7c, the measured *Q* factors are very close by using these two different systems when *Q* factor is small. However, when the *Q* factor of the eigenmodes is larger than 100, the difference between the measured *Q* factors appears and increases with *Q* factor. This is because ZnTe THz-TDS has a longer scanning time (1734 ps) than fiber-based THz-TDS (700 ps), and hence has a smaller spectral resolution. For an eigenmode with a high intrinsic *Q* factor, the information of the resonance beyond 700 ps in the time domain is not captured by the fiber system and the spectral resolution is not small enough to retrieve the whole feature of the resonance, resulting in a low measured *Q* factor. Hence, the measured *Q* factors obtained by ZnTe THz-TDs are closer to the simulated ones.

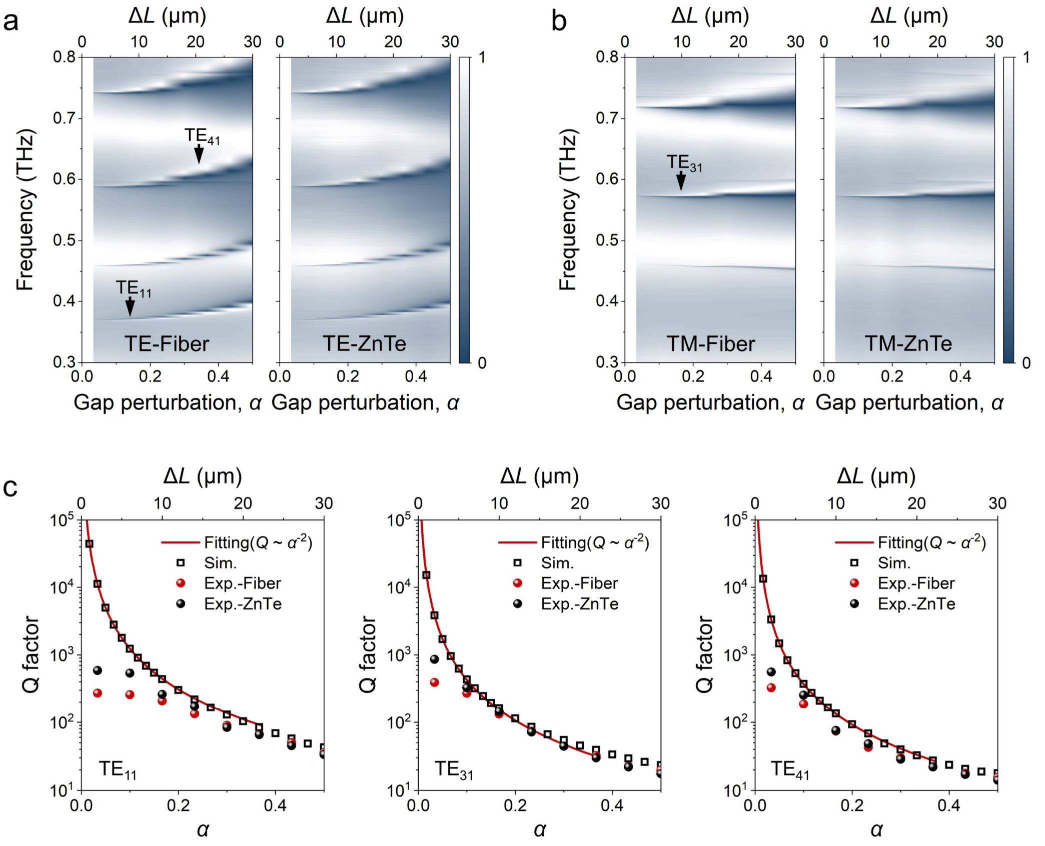

Fig. S7. Transmission spectra and *Q* factors obtained by using two independent terahertz time domain spectrometers (THz-TDS): fiber-based and ZnTe-based THz-TDS. Measured transmission spectra of THz-PhCs with different gap perturbations under normal incidence by using fiber-based (left panel) and ZnTe (right panel) THz-TDSs for a, TE and b, TM polarizations. c, Simulated and measured *Q* factors of GRs TE_{11}, TE_{31}, and TE_{41} modes by using fiber-based and ZnTe THz-TDSs. The simulated *Q* factor is fitted by $Q \propto 1/\alpha^2$ (solid line).

Supplementary Section S8: Measured high *Q* factors of terahertz metasurfaces

High *Q* factor measurement in the terahertz frequency is mainly limited by the spectral resolution of the THz measurement system. Thus, it's very difficult to obtain a *Q* factor higher than 500 in THz metasurfaces even though the intrinsic *Q* factor can approach infinity. Fig. S8a shows the measured high *Q* factors of prominent experimental works found in the current literature[11-22]. Notably, the *Q* factors measured in our work are among the highest. We should note that the highest *Q* factor of 1049 in the reported

terahertz metasurfaces was measured by a THz frequency-domain spectroscopy system[11], whose spectral resolution is 0.14 GHz. It is much smaller than the resolution of our system, which is 0.58 GHz. The measured transmission of the three GRs with the highest Q factors are shown in Fig. S8b, which are well fitted by Eqs. 4-6.

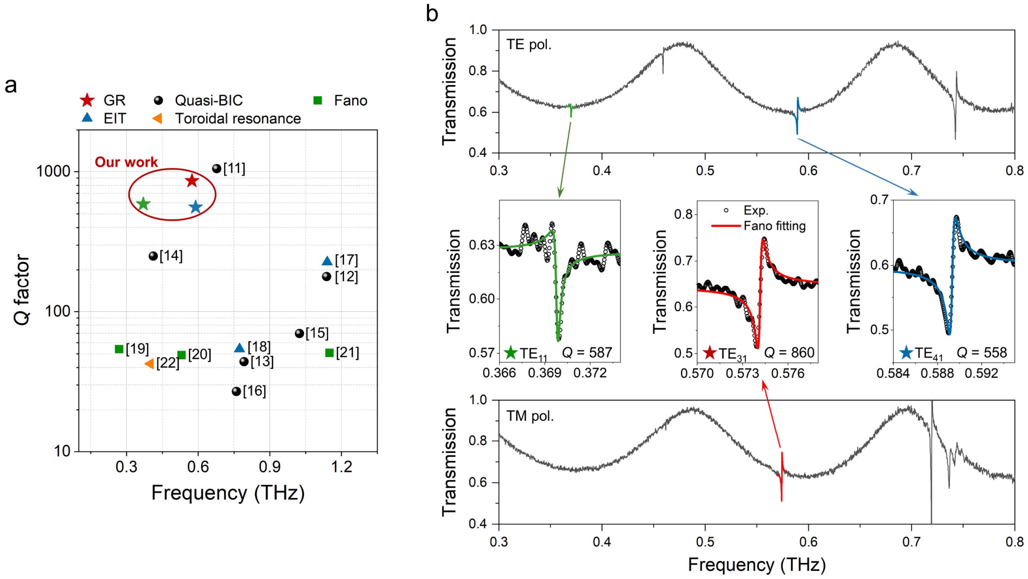

Fig. S8. High *Q* factors in THz metasurfaces. a, Q factors of prominent experimental works found in the current literature as compared to our work. They are classified into five categories according to the mechanisms of the resonant modes: GR, quasi-BIC, Fano resonance, electromagnetically induced transparency (EIT), and toroidal resonance. b, Measured transmission spectra of gap-perturbed THz-PhC (ΔL = 2 μm) for TE and TM polarizations. The target GRs are fitted by the Fano formula.

Supplementary Section S9: Limitations of measurement setup

A maximum Q factor of 860 was measured in this work. However, higher Q factor measurement is difficult due to the following limitations: 1) the resolution of our terahertz spectroscopy is 0.58 GHz, which implies that the maximum Q factor that we could measure is ~1000 if we consider a resonant frequency of 0.6 THz; 2) the diameter of the terahertz beam spot is 8 mm, so the excited mode has a finite lateral size of $L \approx$

8 mm. This finite-sized mode consists of a spread of k points with $\delta k_{\mathrm{mode}} \approx 2\pi/L \approx 3.5\times10^{-2}$ $(2\pi/a_1)$; 3) the terahertz beam has a convergence angle $\theta \approx 6°$, so the source also has a spread of k points with $\delta k_{\mathrm{source}} \approx (2\pi/\lambda)\ \sin(\theta) \approx 5.2\times10^{-2}$ $(2\pi/a_1)$. As shown in Fig. S9a, quasi-BZF-BIC TE_{51} mode of THz-PhC (ΔL = 22 μm) is observed at normal incidence (black line), indicating that the normal incident terahertz beam carries non-zero k components. The measured radiative loss will be the averaged value within this spread of k points. In addition, as the incident angle increases, the measured TE_{31} mode's amplitude decreases due to the degraded collection efficiency of the measurement setup at oblique incidences. It leads to a large discrepancy between the measured and simulated Q factors at large incident angles (Fig. S9c and S9d).

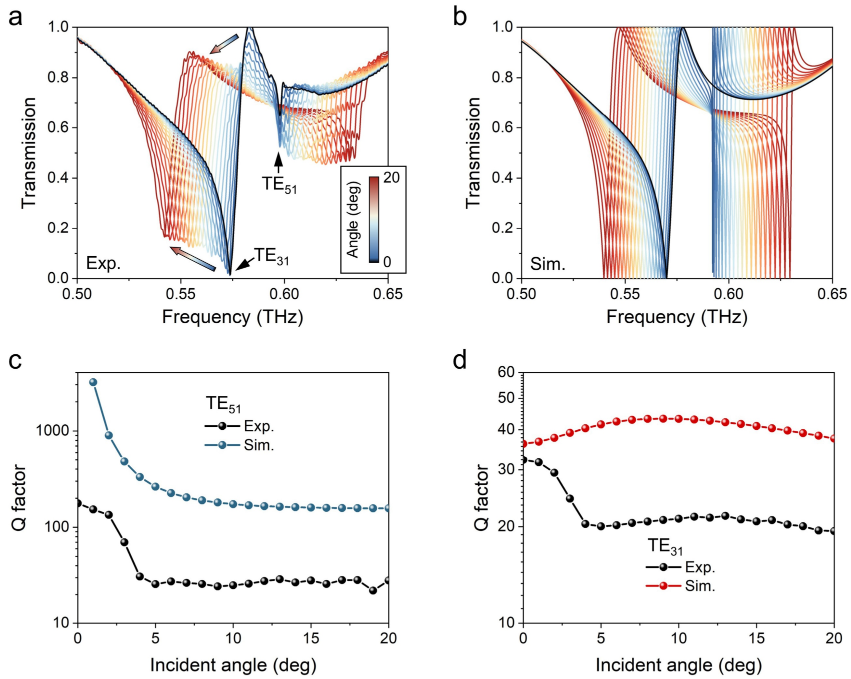

Fig. S9. The discrepancy between measured and simulated results. a, Measured and b, simulated transmission spectra of THz-PhC (ΔL = 22 μm) at different incident angles. Measured and simulated Q factors of c, TE_{51} and d, TE_{31} mode.

Supplementary References

[1] A. C. Overvig, S. Shrestha, N. Yu, *Nanophotonics* **2018**, 7, 1157.

[2] S. Fan, W. Suh, J. D. Joannopoulos, *J. Opt. Soc. Am. A* **2003**, 20, 569.

[3] J. Jin, X. Yin, L. Ni, M. Soljačić, B. Zhen, C. Peng, *Nature* **2019**, 574, 501.

[4] L. Yuan, Y. Y. Lu, *Physical Review A* **2020**, 101, 043827.

[5] B. Zhen, C. W. Hsu, L. Lu, A. D. Stone, M. Soljačić, *Phys. Rev. Lett.* **2014**, 113, 257401.

[6] Y. Zhang, A. Chen, W. Liu, C. W. Hsu, B. Wang, F. Guan, X. Liu, L. Shi, L. Lu, J. Zi, *Phys. Rev. Lett.* **2018**, 120, 186103.

[7] T. Yoda, M. Notomi, *Phys. Rev. Lett.* **2020**, 125, 053902.

[8] J. D. Joannopoulos, S. G. Johnson, J. N. Winn, R. D. Meade, *Photonic Crystals: Molding the Flow of Light*, Princeton University Press, **2008**.

[9] J. Lee, B. Zhen, S.-L. Chua, W. Qiu, J. D. Joannopoulos, M. Soljačić, O. Shapira, *Phys. Rev. Lett.* **2012**, 109, 067401.

[10] K. Sakoda, *Optical properties of photonic crystals*, Springer Science & Business Media, **2004**.

[11] P. Wang, F. He, J. Liu, F. Shu, B. Fang, T. Lang, X. Jing, Z. Hong, *Photon. Res.* **2022**, 10, 2743.

[12] L. Cong, R. Singh, *Adv. Opt. Mater.* **2019**, 7, 1900383.

[13] D. Liu, X. Yu, F. Wu, S. Xiao, F. Itoigawa, S. Ono, *Opt. Express* **2021**, 29, 24779.

[14] S. Han, L. Cong, Y. K. Srivastava, B. Qiang, M. V. Rybin, A. Kumar, R. Jain, W. X. Lim, V. G. Achanta, S. S. Prabhu, Q. J. Wang, Y. S. Kivshar, R. Singh, *Adv. Mater.* **2019**, 31, 1901921.

[15] S. Han, P. Pitchappa, W. Wang, Y. K. Srivastava, M. V. Rybin, R. Singh, *Adv. Opt. Mater.* **2021**, 9, 2002001.

[16] X. Zhao, C. Chen, K. Kaj, I. Hammock, Y. Huang, R. D. Averitt, X. Zhang, *Optica* **2020**, 7, 1548.

[17] W. Cao, R. Singh, I. A. I. Al-Naib, M. He, A. J. Taylor, W. Zhang, *Opt. Lett.* **2012**, 37, 3366.

[18] T. Ma, Q. Huang, H. He, Y. Zhao, X. Lin, Y. Lu, *Opt. Express* **2019**, 27, 16624.

[19] G. Scalari, C. Maissen, S. Cibella, R. Leoni, J. Faist, *Appl. Phys. Lett.* **2014**, 105, 261104.

[20] Y. K. Srivastava, M. Manjappa, L. Cong, W. Cao, I. Al-Naib, W. Zhang, R. Singh, *Adv. Opt. Mater.* **2016**, 4, 457.

[21] Y. P. Cao, Y. Y. Wang, Z. X. Geng, J. Liu, Y. P. Yang, H. D. Chen, *Journal of Applied Physics* **2015**, 117, 063107.

[22] M. Gupta, V. Savinov, N. Xu, L. Cong, G. Dayal, S. Wang, W. Zhang, N. I. Zheludev, R. Singh, *Adv. Mater.* **2016**, 28, 8206.
